# Elevated Serum Amyloid A Is Associated With Cognitive Impairment in Ischemic Stroke Patients

**DOI:** 10.3389/fneur.2021.789204

**Published:** 2022-01-17

**Authors:** Yun Zhang, Yue Feng, Jiacai Zuo, Jian Shi, Shanshan Zhang, Yao Yang, Shu Xie, Zhonglun Chen

**Affiliations:** ^1^Department of Neurology, Mianyang Central Hospital, Mianyang, China; ^2^Department of Medical Laboratory, Mianyang Central Hospital, Mianyang, China

**Keywords:** serum amyloid A, ischemic stroke, cognitive impairment, predictive factor, neuropsychology

## Abstract

**Background and Purpose:**

The impact of serum amyloid A on cognitive impairment after ischemic stroke is unclear. We aimed to investigate the association between serum amyloid A (SAA) levels and post-stroke cognitive impairment (PSCI) at 3 months after ischemic stroke.

**Methods:**

One hundred and ninety-eight patients were enrolled prospectively from June 2020 to April 2021. The SAA concentrations were measured using a commercially available enzyme-linked immunosorbent assay kit after admission. Cognitive function was assessed using the Montreal Cognitive Assessment score at 3 months after the symptom onset. We defined a Montreal Cognitive Assessment score <25 as cognitive impairment.

**Results:**

During 3-month follow-up, 80 patients (40.4%) were diagnosed as having PSCI. As compared with patients with cognitively normal ischemic stroke, those with PSCI were older, more likely to have diabetes and white matter lesions, and had a higher baseline National Institutes of Health stroke score and SAA levels. After adjustment for age, the National Institutes of Health stroke score and other covariates, the OR for the highest quartile of SAA compared with the lowest quartile was 5.72 (95% CI, 2.17–15.04, *P* = 0.001) for PSCI. Also, ordinal logistic regression analysis showed that higher SAA concentrations were associated with increased risk of PSCI severity (OR, 4.31; 95% CI, 1.81–10.33, *P* = 0.001). Similar results were found when the SAA levels were analyzed as a continuous variable.

**Conclusions:**

This present study demonstrated that increased SAA levels might be associated with PSCI at 3 months after ischemic stroke.

## Introduction

Stroke is one of the major devastating diseases with high rates of mortality and long-term disability (1, 2). Post-stroke cognitive impairment (PSCI) is a common consequence of stroke affecting as high as 80% of stroke survivors (3, 4). It may reduce medication adherence and induce a poor functional outcome after stroke (5–7). Also, it has been reported that PSCI may be associated with increased risk of mortality and recurrent stroke (6, 8, 9). To date, associated factors and underlying pathophysiology of PSCI are not well-determined, which may be a contributing factor to why the mortality and long-term disability risk in ischemic stroke survivors remained high. Therefore, determining new prognostic markers is of vital importance for continuously improving the prognosis of stroke and providing insight into underlying pathophysiology of PSCI.

Serum amyloid A is one of highly conserved acute-phase proteins, which are involved in the chemotactic recruitment of inflammatory cells (10). Serum amyloid A (SAA) concentrations rise rapidly when the body is infected, wounded, or inflamed. The production of acute-phase SAA is triggered by proinflammatory cytokines, such as interleukin-1, interleukin-6, and transforming growth factor-β. Recently, it has been proved that SAA may play an important role in various central nervous system diseases (10, 11). SAA has been found to be substantially associated with the severity of senile early cognitive impairment (12). In a recent experimental study, cerebral ischemia could induce a systemic inflammatory response through SAA that might contribute to the pathological outcomes (13). Also, previous studies reported that SAA levels were significantly higher in patients with ischemic stroke who died compared with those who survived and were independently associated with early death, after adjusting for various confounders (14). However, in the cognitive impairment after ischemic stroke, the role of SAA has not yet been assessed. In this prospective cohort study, we aim to assess whether SAA levels could be an effective predictor of PSCI during a 3-month follow-up period.

## Materials and Methods

### Study Participants

Patients with first ever ischemic stroke were prospectively recruited from June 2020 to April 2021 in Mianyang Central Hospital. The patients presenting with acute ischemic stroke were eligible if they were older than 18 years and were admitted within 7 days of the symptom onset. The exclusion criteria were as follows: (1) history of a central nervous system disease such as Alzheimer's disease, Parkinson's disease, trauma, multiple sclerosis, and central nervous system infection; (2) history of cognitive dysfunction, psychiatric illness, and substance abuse/dependence before the index stroke; (3) pneumonia or other infectious diseases prior to the ischemic stroke onset within 1 month; (4) life expectancy <3 months. We also excluded the patients that could not complete the cognitive function examination.

### Baseline Data Collection

Clinical data were recorded after admission as follows: demographic characteristics, vascular risk factors, such as hypertension, diabetes mellitus, and hyperlipidemia; body mass index (BMI); blood pressure; stroke severity; and etiology of stroke. Stroke severity was evaluated using National Institutes of Health Stroke Scale (NIHSS) (15). Stroke subtypes were analyzed by the TOAST (Trial of Org 10172 in Acute Stroke Treatment) criteria, including large-artery atherosclerosis, cardioembolism, small vessel disease, others, and unknown (16). We performed laboratory tests, including fasting blood glucose, C-reactive protein levels (CRP), total cholesterol, triglyceride, low-density lipoprotein cholesterol, and high-density lipoprotein cholesterol.

### Measurement of SAA Levels

For measuring serum SAA levels, venous blood samples were obtained within 24 h after admission. The specimens were immediately separated by centrifugation at 1,200 rpm for 15 min and the isolated serum frozen at −80°C for further analysis. The SAA concentrations were detected using the commercially available enzyme-linked immunosorbent assay kit (Invitrogen Corp). All assay procedures were conducted according to the manufacturer's instructions by experienced laboratory technicians who did not have prior knowledge of the subjects' clinical data.

### Assessment of PSCI

The cognitive functions were assessed at 3 months after stroke by qualified neurologists, who were blind to the clinical data, using the Montreal Cognitive Assessment (MoCA) (17). MoCA has been translated into Chinese and validated as an efficient screening tool for cognitive impairment in the Chinese population. To correct for education effects, 1 point was added in the total MoCA score if the patients were with education <12 years. In this study, cognitive impairment was defined as a MoCA score of <25 (18–20). Furthermore, the severity of PSCI was categorized as follows: a MoCA score of 25–30 (no cognitive impairment), 20–24 (mild cognitive impairment), and 0–19 (severe cognitive impairment) (20).

### Statistical Analysis

Continuous variables were expressed as mean ± standard deviation (SD) if normally distributed or median (interquartile range, IQR) if not. Normally distributed variables were analyzed with *t*-test, and abnormal distributed variables were compared with Mann–Whitney *U*-test. Categorical data were demonstrated as percentages and compared by χ^2^ test or Fisher's exact test. Binary logistic regression and ordinal logistic regression were used to assess the association between serum SAA and clinical outcomes when appropriate. All multivariable analyses were first adjusted for age and sex (Model 1) and additionally adjusted for all variables with *P* <0.1 in univariate analysis (including age, sex, diabetes mellitus, white matter lesions, baseline NIHSS score, and CRP levels; Model 2). OR and 95% CI were calculated for higher quartiles of SAA compared with the lowest quartile. Linear regression analysis was further used to explore the association between serum SAA and the total MoCA score. Also, we calculated the net reclassification index (NRI) and integrated discrimination improvement (IDI) to characterize the improvement in predictive performance when the SAA levels were added to a conventional model. The NRI and IDI are statistics proposed as measures of the increasing prognostic impact that a new marker will have when added to an existing prediction model for a binary outcome. All analyses were performed using SPSS version 24.0 (SPSS Inc., Chicago, IL, USA) and R language (version 4.0.4). All tests were based on 2-tailed, and statistical significance was set at *P* < 0.05.

## Results

Finally, 198 patients (mean age, 65.7 ± 9.9 years; 58.1% male) with first-ever ischemic stroke were enrolled in this study. Among these patients, 66.2% had hypertension, 25.3% had diabetes mellitus, 15.2% had hyperlipidemia, and 35.9% had white matter lesions. During the 3-month follow-up, 80 patients (40.4%) experienced cognitive impairment. Demographic and clinical characteristics between the patients with and without PSCI are presented in Table 1. In univariate analysis, the prevalence of diabetes mellitus in patients with PSCI was higher than in patients without PSCI (36.3 vs. 17.8%; *P* = 0.003). Patients with PSCI were older than those without it (mean, 67.6 ± 9.1 years vs. 64.5 ± 10.2 years; *P* = 0.031). White matter lesion was more prevalent in the patients with PSCI than in the patients without it (45 vs. 29.7%; *P* = 0.027). Also, the patients with PSCI had a high baseline NIHSS score [median 6 (2, 8) score vs. 4 (2, 6) score; *P* = 0.030] and SAA levels [median, 60.1 (44.9, 76.3) mg/L vs. 50.5 (35.9, 64.5) mg/L, *P* = 0.002].

**Table 1:** Baseline data according to patients with and without cognitive impairment.

**Variables**	**Total population (*n* = 198)**	**With PSCI (*n* = 80)**	**Without PSCI (*n* = 118)**	*P-*value^*^
Demographic characteristic				
Age, years, mean ± SD	65.7 ± 9.9	67.6 ± 9.1	64.5 ± 10.2	0.031
Male, *n* (%)	115 (58.1)	41 (51.3)	74 (62.7)	0.119
Clinical data				
Time from onset to admission, days, median (IQR)	2.0 (1.0, 3.0)	2.0 (2.0, 4.0)	2.0 (1.0, 3.0)	0.292
Hypertension, *n* (%)	131 (66.2)	56 (70.0)	75 (63.6)	0.347
Diabetes mellitus, *n* (%)	50 (25.3)	29 (36.3)	21 (17.8)	0.003
Hyperlipidemia, *n* (%)	30 (15.2)	14 (17.5)	16 (13.6)	0.448
Coronary heart disease, *n* (%)	27 (13.6)	9 (11.3)	18 (15.3)	0.420
Current smoker, *n* (%)	87 (43.9)	34 (42.5)	53 (44.9)	0.737
Systolic blood pressure, mmHg, mean ± SD	138.1 ± 16.9	137.8 ± 16.3	138.2 ± 17.3	0.853
Diastolic blood pressure, mm Hg, mean ± SD	81.7 ± 10.2	81.9 ± 10.1	81.6 ± 10.4	0.863
BMI, kg/m^2^, mean ± SD	24.7 ± 3.3	24.8 ± 3.0	24.7 ± 3.1	0.760
Baseline NIHSS, median (IQR)	5.0 (2.0, 7.0)	6.0 (2.0, 8.0)	4.0 (2.0, 6.0)	0.024
White matter lesions, *n* (%)	71 (35.9)	36 (45.0)	35 (29.7)	0.027
Stroke subtypes				0.209
Large-artery atherosclerosis, *n* (%)	90 (45.5)	34 (42.5)	56 (47.5)	
Cardioembolism, *n* (%)	40 (20.2)	20 (25.0)	20 (16.9)	
Small vessel disease, *n* (%)	54 (27.3)	18 (22.5)	36 (30.5)	
Others or unknown, *n* (%)	14 (7.1)	8 (10.0)	6 (3.0)	
Laboratory data				
C-reactive protein, mg/L, median (IQR)	5.2 (2.6, 9.6)	7.0 (2.4, 10.5)	4.6 (2.6, 7.7)	0.075
Blood glucose, mmol/L, mean ± SD	5.9 ± 2.5	5.8 ± 2.1	6.0 ± 2.7	0.390
Homocysteine,μmol/L, mean ± SD	14.3 ± 6.4	13.3 ± 5.2	13.9 ± 4.9	0.443
Total cholesterol, mmol/L, mean ± SD	4.0 ± 1.0	4.0 ± 1.1	3.9 ± 1.0	0.626
Triglyceride, mmol/L, median (IQR)	1.4 (1.1, 1.8)	1.5 (1.1, 1.9)	1.3 (1.1, 1.7)	0.225
HDL, mmol/L, mean ± SD	1.1 ± 0.2	1.1 ± 0.2	1.1 ± 0.2	0.426
LD, mmol/L, median (IQR)	2.4 (2.0, 2.9)	2.3 (1.9, 2.9)	2.5 (2.0, 3.0)	0.195
Serum amyloid A, mg/L, median (IQR)	52.4 (39.2, 68.8)	60.1 (44.9, 76.3)	50.5 (35.9, 64.5)	0.002
Serum amyloid A quartile				0.006
First quartile	47 (23.7)	11 (13.8)	36 (30.5)	
Second quartile	52 (26.3)	19 (23.8)	33 (28.0)	
Third quartile	51 (25.8)	22 (27.5)	29 (24.6)	
Fourth quartile	48 (24.2)	28 (35.0)	20 (16.9)	

*BMI, body mass index; HDL, high-density lipoprotein cholesterol; IQR, interquartile range; LDL, low-density lipoprotein cholesterol; SD, standard deviation*.

* *The P-value was calculated by comparing the differences between patients with and without post-stroke cognitive impairment*.

**Figure 1:**
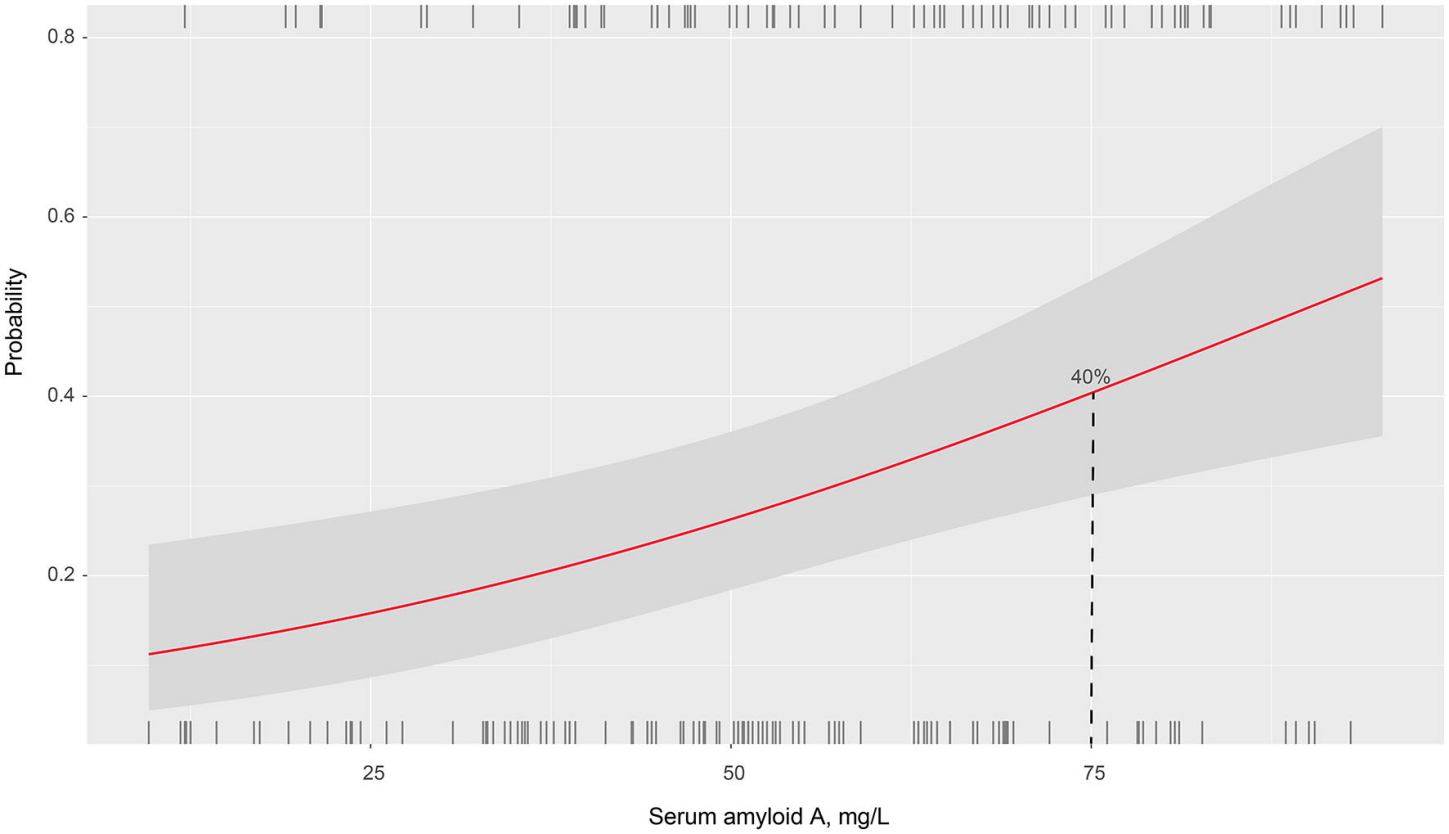
Association of serum amyloid A levels with the probability of post-stroke cognitive impairment in patients with ischemic stroke.

The prevalence of PSCI at 3 months in 4 quartiles of serum amyloid A levels (from low to high) were 13.8, 23.8, 27.5, and 35%, respectively. After adjustment for potential confounders in binary logistic regression analysis, the OR of cognitive impairment associated with the highest SAA quartile was 5.72 (95% CI, 2.17–15.04; *P* = 0.001). On continuous scale, each SD increase of SAA was associated with 55% (95% CI, 1.16–2.01) increased risk of PSCI (Table 2). We found that the predicted probabilities for PSCI were >40% after 75 mg/L of SAA (Figure 1). Adding SAA levels to a model containing conventional risk factors significantly improved risk reclassification for PSCI (category-free net reclassification index, 0.232, 95% CI, 0.081–0.323, *P* = 0.003; integrated discrimination improvement, 0.048, 95% CI, 0.017–0.079, *P* = 0.000) (Table 3).

**Table 2:** Logistic regression analysis for the associations between serum amyloid A (SAA) levels and a clinical outcome.

	**Binary logistic regression for PSCI**		**Ordinal regression analysis for PSCI severity**	
	**OR (95% CI)**	***P-*value**	**OR (95% CI)**	***P-*value**
**Model 1**				
SAA (Per SD increase)	1.44 (1.09–1.87)	0.008	1.47 (1.14–1.90)	0.003
SAA quartiles				
First quartile	Reference		Reference	
Second quartile	2.00 (0.82–4.91)	0.129	1.98 (0.92–4.26)	0.082
Third quartile	2.21 (0.91–5.41)	0.081	2.72 (1.25–5.96)	0.012
Fourth quartile	4.94 (1.99–12.25)	0.001	4.40 (1.87–13.83)	0.001
**Model 2**				
SAA (Per SD increase)	1.55 (1.16–2.01)	0.003	1.61 (1.14–2.29)	0.007
SAA quartiles				
First quartile	Reference		Reference	
Second quartile	2.21 (0.83–5.89)	0.112	1.91 (0.88–4.20)	0.104
Third quartile	2.53 (0.97–6.59)	0.057	2.56 (1.14–5.81)	0.022
Fourth quartile	5.72 (2.17–15.04)	0.001	4.31 (1.81–10.33)	0.001

*OR, odds ratio; CI, confidence interval; SAA, Serum amyloid A*.

*Model 1: adjusted for age and sex*.

*Model 2: adjusted for age and sex and variables with P < 0.1 in univariate analysis including diabetes mellitus, white matter lesions, baseline NIHSS score, and CRP levels*.

**Table 3:** Reclassification statistics for cognitive impairment by SAA levels.

**Variables**	**NRI (category free)**		**IDI**	
	**Estimate (95% CI)**	***P-*value**	**Estimate (95% CI)**	***P-*value**
Conventional model^*^	Reference		Reference	
SAA (continuous)	0.232 (0.081–0.323)	0.003	0.048 (0.017–0.079)	0.002
SAA (quartiles)	0.299 (0.144–0.454)	0.001	0.056 (0.023–0.088)	0.001

*CI, confidence interval; SAA, Serum amyloid A*.

* *Conventional model included age, sex, diabetes mellitus, white matter lesions, baseline NIHSS score, and CRP levels*.

**Figure 2:**
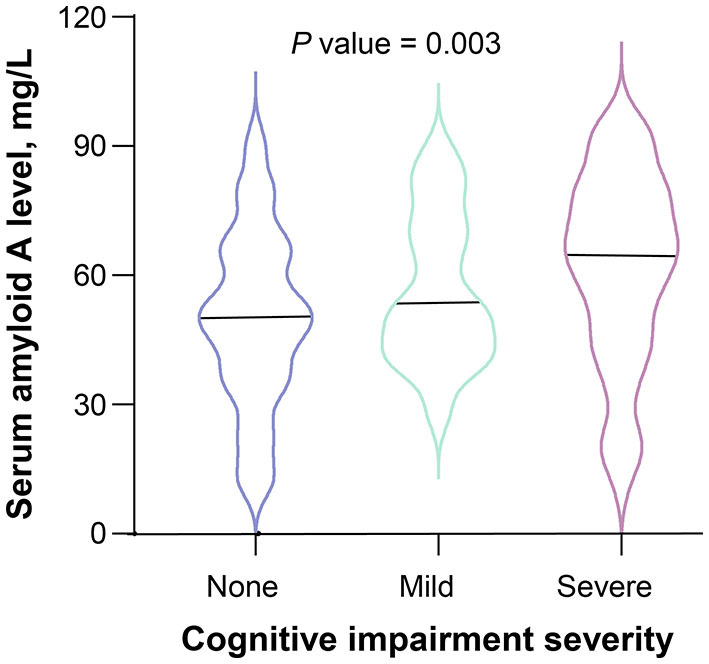
Serum amyloid A levels and severity of cognitive impairment after ischemic stroke. The black horizontal line represents the median. The differences of serum amyloid A levels between the 3 groups of cognitive impairment severity were evaluated using Kruskal-Wallis test.

**Figure 3:**
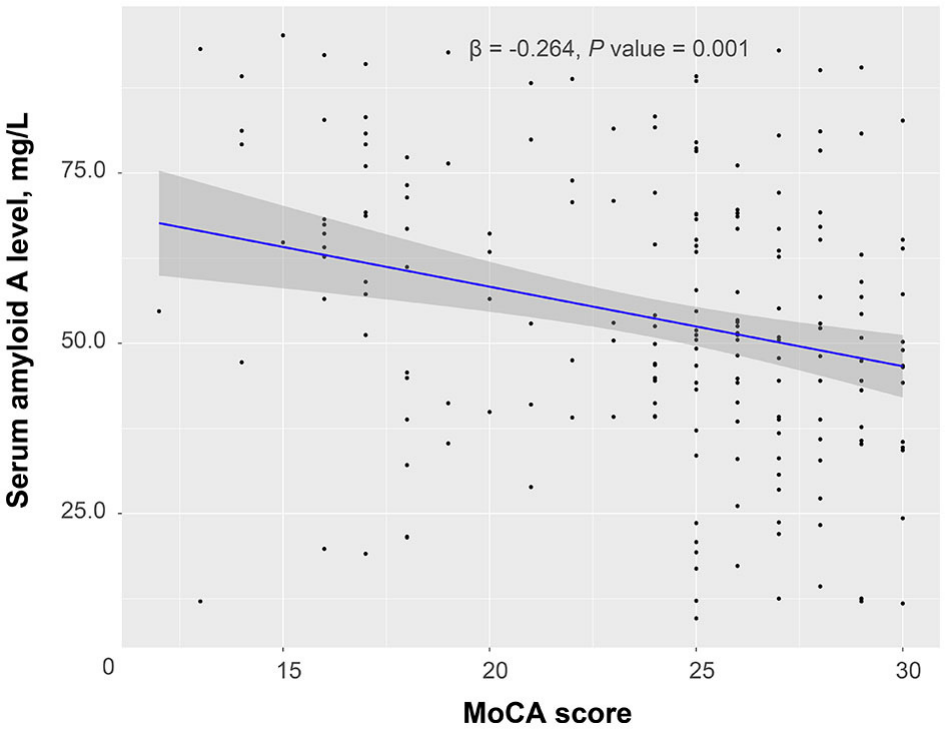
Linear regression analysis confirmed a significant negative association between serum amyloid A levels and a Montreal Cognitive Assessment (MoCA) score.

According to the 3-month MoCA score, a total of 80 patients had PSCI, among which 34 were mild PSCI and 46 were severe PSCI. Univariate analysis confirmed an increased trend of SAA levels across PSCI severity (*P* = 0.003; Figure 2). Multivariable ordinal logistic regression analysis showed that the highest SAA quartile was associated with PSCI severity (as compared with the lowest SAA quartile, OR 4.31, 95% CI, 1.81–10.33; *P* = 0.001). Similar results were found when the SAA levels were analyzed as a continuous variable (per 1-SD increase, OR 1.62, 95% CI 1.14–2.29; *P* = 0.007). Furthermore, a significant negative association was found between SAA levels and the MoCA score by linear regression analysis (standard error = 0.016, β = −0.264, *P* = 0.001; Figure 3).

## Discussion

In the present study, we tested the hypothesis that SAA concentrations were associated with increased risk of cognitive impairment in patients with ischemic stroke. Several key findings were noted in this analysis. First, increased SAA levels significantly associated with higher risk of PSCI at 3 months. Second, we confirmed that the patients with greater PSCI severity had higher SAA levels than the patients with cognitively normal ischemic stroke. Furthermore, these associations remained robust after adjusting for age, sex, traditional risk factors, and baseline neurological deficit.

Post-stroke cognitive impairment (PSCI) is a series of clinical syndromes that meet the diagnostic criteria for cognitive impairment that appears after ischemic stroke. PSCI has become one of the main targets for secondary prevention of stroke. Earlier studies had generally shown an inconsistent result in the prevalence of PSCI (4–6, 21–23); this could possibly be explained by the differences in race, screening tools, and time interval for assessing the cognitive function. In our study, the incidence of PSCI at 3 months after the ischemic stroke onset was 40.4%. Consistent with our findings, the incidence of cognitive impairment was reported to be 37.3% in a study of 209 patients with mild ischemic stroke (23). However, another prospective study recruiting 796 Chinese patients with ischemic stroke demonstrated that the prevalence of PSCI reached its peak (72%) 3 months after the stroke onset and decreased to 30.3% at 12 months (21). These results suggested an urgent need for an internationally agreed definition of PSCI.

Several traditional risk factors were found to be correlated with PSCI in our study, such as diabetes mellitus, white matter lesions, baseline NIHSS score, and CRP levels. Recent surveys have underlined the importance of white matter lesions for cognitive impairment after stroke (24). The patients with more severe white matter lesions are prone to display worsening of cognitive function, which could account for their apparent associations with PSCI. Also, severe white matter lesions are mainly correlated with frontal lobe dysfunction, which was recognized as the consequence of damaged neural transmission and interneural connection (25). Similarly, Geng et al. analyzed the data of a Stroke Registry database and observed that patients with PSCI had severe stroke severity as compared with those without PSCI (21). Therefore, the relationship between SAA and cognitive impairment might be caused by above factors. After adjusting for several predictive factors, including baseline stroke severity and inflammatory markers, SAA remained independently associated with cognitive impairment, suggesting an incremental prognostic value beyond these traditional risk factors.

The mechanisms by which SAA affects PSCI are unclear, but several potential pathophysiological pathways have been proposed. First, as an acute-phase inflammatory mediator, SAA concentrations rise rapidly when the body is infected, wounded, or inflamed (26). Previous studies demonstrated that SAA might activate the NLRP3 inflammasome in the brain by mediating ROS in the microglial cells through the inhibition by NAC and mito-TEMPO, which directly contributes to the damage of neuron and cognitive function (13, 27). Second, both the SAA mRNA and protein expression colocalize with astrocytes and macrophages/microglia in the cortex, corpus callosum, thalamus, and hippocampus after brain injury (28). The location of stroke plays an important role in the development of cognitive impairment. The changes of SAA expression in the limbic cortex and hippocampus might be associated with memory loss and cognitive impairment (29, 30). Other possible pathways include causing damaging the endothelial cells and a blood-brain barrier, increasing oxidative stress, and inducing atherosclerosis (13, 31–33). Further studies are needed to detect whether the management of SAA within appropriate range could improve cognitive function after ischemic stroke.

Several limitations should be noted in this study. First, this is a preliminary study with a small sample from one single stroke center, and all the patients are Chinese. Therefore, these results could not be generalized to all populations. Second, SAA concentrations were tested only one time at the baseline, so we were unable to assess the association between SAA changes and status of cognitive function. Third, several potential confounders, which might affect the cognitive function, were not included in this study, such as location of lesion and lesion volume. Finally, the patients with severe neurological deficit and history of cognitive dysfunction and psychiatric illness were excluded from this study, which might underestimate the actual prevalence of PSCI. These issues should be addressed in future well-controlled multicenter studies with a large sample.

In summary, this study showed that increased SAA levels were associated with cognitive impairment after ischemic stroke, regardless of the initial stroke severity, and other cardiovascular risk factors. Accordingly, SAA can be used as an early efficient predictive factor to stratify patients into high risk or low risk of cognitive impairment in the early acute ischemic stroke stage. Further studies are needed to replicate our findings and provide insight into the role of SAA in PSCI.

## Data Availability Statement

The raw data supporting the conclusions of this article will be made available by the authors, without undue reservation.

## Ethics Statement

The studies involving human participants were reviewed and approved by Ethics Committee of Mianyang Central Hospital. The patients/participants provided their written informed consent to participate in this study. Written informed consent was obtained from the individual(s) for the publication of any potentially identifiable images or data included in this article.

## Author Contributions

YZ and ZC: conceptualization, project administration, supervision, and visualization. YZ and YF: data curation and formal analysis. JZ, JS, SZ, and YY: investigation. YZ, SX, and ZC: methodology. ZC: validation. YZ: writing the original draft. YZ and ZC: writing, review, and editing. All authors contributed to the article and approved the submitted version.

## Conflict of Interest

The authors declare that the research was conducted in the absence of any commercial or financial relationships that could be construed as a potential conflict of interest.

## Publisher's Note

All claims expressed in this article are solely those of the authors and do not necessarily represent those of their affiliated organizations, or those of the publisher, the editors and the reviewers. Any product that may be evaluated in this article, or claim that may be made by its manufacturer, is not guaranteed or endorsed by the publisher.
